# Management of Labral Tears Associated with Glenohumeral Instability in Athletes

**DOI:** 10.1007/s12178-026-10044-9

**Published:** 2026-06-19

**Authors:** Mina Entessari, Azra N Dees, Sarah E Watson, Dyrian D Wandick, Brian R Waterman

**Affiliations:** 1 Atrium Health Wake Forest Baptist, Department of Orthopaedic Surgery and Rehabilitation, 1 Medical Center Blvd, Winston-Salem, NC 27157 USA; 2 Wake Forest University School of Medicine, Winston-Salem, NC USA

**Keywords:** Labral Tears, Shoulder Instability, Rehabilitation, Return to Sport

## Abstract

**Purpose of Review:**

Glenoid labral tears account for more than 10% of surgically managed shoulder injuries and are classified and managed depending on location, mechanism, associated bone loss, and patient-specific risk factors. The purpose of this review is to examine the current literature on the evaluation and management of labral pathology with associated glenohumeral instability, with a focus on rehabilitation and return to sport.

**Recent Findings:**

While the management of anterior labral tears has historically been emphasized, recent studies demonstrate that posterior and combined capsulolabral injuries are more common than previously recognized. Advances in management include reduced time to surgical treatment, lower thresholds of critical glenohumeral bone loss, evolving surgical techniques with both bony and soft tissue reconstruction, as well as personalized rehabilitation protocols with a focus on periscapular and rotator cuff strengthening. Operative stabilization demonstrates superior outcomes compared to nonoperative treatment, especially in high-risk patients (e.g young age, contact sport participation, hyperlaxity). However, recurrent instability remains a persistent problem, necessitating a comprehensive approach to management in order to optimize outcomes.

**Summary:**

Optimal functional outcomes in managing labral tears require a multifaceted, patient-specific approach. Treatment decisions, rehabilitation protocols, and return to sport testing should consider individual risk factors, account for combined pathology and bone loss, and consider psychological readiness to prevent recurrent instability and optimize long-term outcomes.

## Introduction

Glenoid labral tears are a common subset of shoulder injuries, accounting for more than 10% of surgically treated shoulder pathology [1, 2]. While historical literature suggested that up to 90% of labral tears are anterior, more recent data indicate this may be outdated. Current estimates indicate that up to 47% of labral injuries are purely posterior, and more than 75% involve combined anterior-posterior pathology [1, 3, 4]. Approximately 60% of these tears are traumatic, with 31.4% associated with dislocation events [1].

So-called clinically significant labral injuries are categorized by location, mechanism, and anatomic position on the clockface, with each of these variables associated with distinct clinical implications [5]. Bankart lesions are the most common type associated with anterior instability, involving the anteroinferior glenohumeral ligament with capsular disruption that may be bony or soft tissue [6]. Non-Bankart lesions include anterior labroligamentous periosteal sleeve avulsion (ALPSA), glenolabral articular disruption (GLAD), and Perthes lesions—each with subtle, but characteristic structural features [6, 7]. Superior labral anterior to posterior (SLAP) tears involve the superior labrum and biceps anchor and are common in overhead athletes and laborers [8]. Conversely, posterior tears are increasingly recognized, particularly in collision athletes and military populations, where instability incidence is 2–8 times greater than in civilian counterparts [9]. A summary of labral lesions is provided in Table 1.

Clinical presentation varies by tear location. Posterior tears more commonly present with complaints of pain (68%) than instability (22%), whereas anterior tears frequently present with instability (62.5%) rather than overt shoulder pain (21%) [1]. Superior tears often present with deep shoulder pain provoked by overhead activity [10]. Multidirectional instability (MDI) is typically atraumatic and aggravated due to repetitive microtrauma. MDI presents with instability in more than one direction, one of which is inferior due to an insufficient inferior capsule involving the anterior and posterior bands of the inferior glenohumeral ligament (IGHL) as well as rotator interval deficiency. Patients with MDI often have associated labral pathology due to repeated subluxation and dislocation events [11]. Notably, labral abnormalities are present in up to 9% of asymptomatic non-athletic adults on magnetic resonance imaging (MRI) and can be as high as 30–75% in selected subsets, emphasizing the importance of correlating imaging with clinical findings [2, 12, 13]. Combined injuries are nearly twice as common as isolated tears and may influence both presentation and management [14].

Management strategies continue to evolve. Advances in suture anchor technology alongside bony and soft tissue stabilization techniques have improved clinical outcomes, though nonoperative management remains appropriate in select patients [15]. Rehabilitation protocols for nonoperative and operative management of labral pathology focus on initial immobilization, reduction of pain, restoration of range of motion, and strengthening of periscapular and rotator cuff muscles. While overlap in rehabilitation protocols exists, differences in timelines and sport-specific training are based on patient age, activity level, pathology, and surgical procedures performed (bony versus soft tissue only). The goal of this review is to provide a comprehensive overview of glenoid labral tears associated with glenohumeral instability in athletic patients, with a focus on management, rehabilitation, and outcomes.

**Table 1:** Summary of Labral Tears

Type of Tear	Location (right shoulder clock face description)	Description
Bankart	Anteroinferior (3–6 o’clock)	Avulsion of anteroinferior labrum from glenoid rim with capsular disruption; can be soft tissue or bony (fracture of anteroinferior glenoid rim) [6]
ALPSA	Anteroinferior (2–5 o’clock)	Anteroinferior labrum torn off from glenoid with an intact but medially displaced periosteum; labrum heals medially and inferiorly rotated on glenoid neck; recurrence rates 2x higher than Bankart (8.3–19.2%) due to loss of periosteal hinge on the glenoid neck [6, 7]
GLAD	Anteroinferior (3–5 o’clock)	Superficial tear of anteroinferior labrum with adjacent glenoid articular cartilage injury; labrum remains attached; uncommon; presents with pain but no instability [6, 7]
Perthes	Anteroinferior (3–6 o’clock)	Nondisplaced labral avulsion with intact periosteum; labrum remains in anatomic position but is detached from glenoid; often missed on standard imaging [6, 7]
SLAP	Superior (10 − 2 o’clock)	Tear of superior labrum involving biceps anchor most commonly from direct force to abducted arm; Types I-X based on degree of involvement; prevalent in overhead, contact athletes and military populations [8]
Posterior	Posteroinferior (7–11 o’clock)	Avulsion of posteroinferior labrum; presents predominantly with pain (68%) rather than instability (21%); increasingly recognized in military and contact athlete populations; incidence 2-8x higher in military than civilian populations [1, 9]
Kim	Posteroinferior (6–9 o’clock)	Subset of posterior lesions; incomplete, deep tear of junction between the posteroinferior labrum and glenoid with intact superficial labrum; high prevalence in young, active, and overhead throwing populations; often missed without arthroscopic evaluation [16]

*ALPSA *Anterior labroligamentous periosteal sleeve avulsion, *GLAD *Glenolabral articular disruption, *SLAP *Superior labrum anterior to posterior

## Anatomy and Biomechanics

### Glenohumeral Joint Anatomy and Biomechanics

The glenohumeral joint is often described as a “golf ball on a tee,” reflecting the mismatch between the large humeral head and shallow glenoid with limited bony constraint [17]. This anatomy allows for the greatest range of motion in the body with six degrees of freedom, while also predisposing the joint to instability when homeostasis is compromised. Stability is therefore dependent on the effectiveness of both static and dynamic stabilizers.

### Static Stabilizers

Static stabilizers include the labrum, glenohumeral ligaments, joint capsule, bony articulation, and negative intra-articular pressure [75]. The labrum deepens the glenoid and serves as an attachment site for ligaments and the long head of the biceps [10, 17]. The superior, middle, and inferior glenohumeral ligaments function as primary restraints depending on arm position [18]. The IGHL complex, including anterior and posterior bands and the axillary pouch, is critical in maintaining stability at 90° of abduction [18]. Anatomic variants, such as the Buford complex, labral foramen, hypoplastic glenoid, or meniscoid labrum with sublabral recess, may subtly alter glenohumeral function and should be recognized during systematic evaluation [10].

### Dynamic Stabilizers

Dynamic stabilizers include the rotator cuff, periscapular musculature, and long head of the biceps [17]. These structures are important in mid-range motion, where capsuloligamentous structures are relatively lax. The rotator cuff centers the humeral head within the glenoid, maintaining joint congruency through concavity compression [17].

### Blood Supply

The labrum is supplied primarily by the suprascapular, circumflex scapular, and posterior circumflex humeral arteries [10]. The posterosuperior and inferior regions are more vascular than the anterosuperior region, which has important implications for healing.

## Clinical Presentation and Diagnosis

**Fig. 1:**
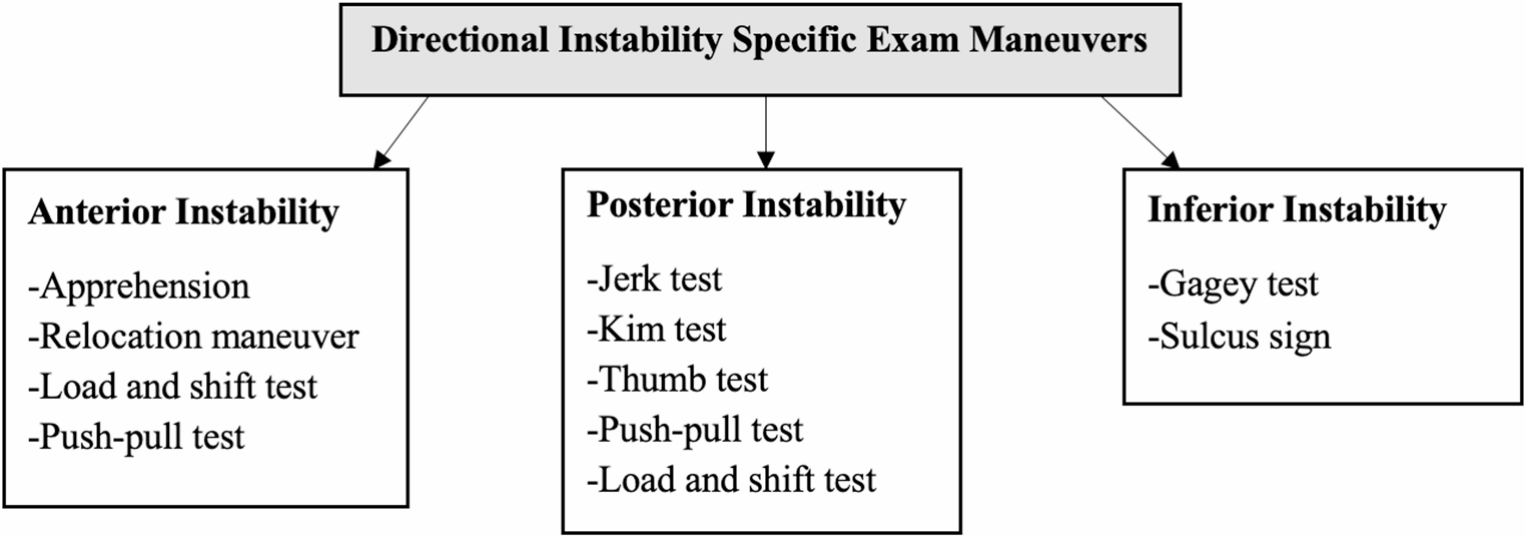
Flowchart reviewing physical exam maneuvers for shoulder instability

Patients with labral pathology often report vague, deep shoulder pain with mechanical symptoms such as clicking, catching, or frank instability [19, 20]. Anterior instability typically occurs with abduction and external rotation, while posterior instability often results from repetitive microtrauma or a direct, posteriorly directed force in flexion and internal rotation [10, 19–21]. Inferior instability may present during load-bearing activities [11]. In cases of MDI, it is important to assess hypermobility using Beighton’s criteria, as; patients with generalized ligamentous laxity possess a 2.5 times higher risk for MDI [11]. Provocative exam maneuvers are summarized in Fig. 1.

## Imaging

Initial evaluation includes standard radiographs, including anteroposterior (AP), Grashey, scapular Y, and axillary views [21, 22]. Specialized radiographs include the West Point view, to visualize bony Bankart lesions, the Stryker notch view to visualize Hill-Sachs lesions, and the Bernageau view to assess attritional bone loss. MRI is the preferred modality for evaluating soft tissue injury, while Computed Tomography (CT) is optimal for assessing glenoid bone loss, particularly with three-dimensional (3D) *en face* reconstructions with humeral head subtraction [17]. 3D reconstructions of the glenoid allow for more accurate measurement of the glenoid track, which is reported to be 84% of the inferior glenoid width [23]. Furthermore, characterization of the Hill-Sachs lesion should be performed to quantify the distance to dislocation and cephalad-caudad position, shown to be critical in determining the subsequent risk for recurrent instability [24, 25]. Magnetic resonance arthrograms are useful in chronic or recurrent cases to assess capsular integrity, diagnosing humeral avulsion of the glenohumeral ligament (HAGL) lesions, whereas CT arthrograms are less sensitive [10].

## Management

The management of the first-time anterior (and posterior) shoulder instability events remains controversial. The shift towards earlier surgical treatment of labral pathology has been reported, coupled with expanded indications for operative management and evolving surgical techniques. When deciding between operative and nonoperative management, risk factors for recurrence, including participation in high-collision or contact sports, male sex, age 16–25, and arm dominance, must be considered [26]. Recurrent instability leads to progressive soft tissue compromise, chondral damage, and attritional bone loss, further complicating management and impacting the overall health and longevity of the joint. Meanwhile, shorter time from injury to surgery has been associated with lower recurrence rates and improved patient outcomes [27].

In a 2020 Delphi study by the Neer circle of American Shoulder and Elbow Surgeons on the treatment of first-time anterior glenohumeral dislocation, > 90% consensus was reported on strong recommendations for surgery in contact athletes aged > 14 at the end of their season with apprehension and meaningful bone loss. Alternatively, there was > 90% consensus on strong recommendations for nonoperative management after first-time anterior shoulder dislocation in non-athletes lacking apprehension without meaningful bone loss [26].

### Nonoperative Management

Trials of nonoperative management consist of activity modification, rehabilitation, and a period of immobilization with bracing, typically ranging from 2 to 6 weeks. There is limited evidence supporting bracing in the prevention of recurrence, though it may provide symptomatic relief [28, 29]. Functional bracing aims to avoid the “at-risk” position of dislocation, specifically abduction and external rotation for anterior instability, but may restrict range of motion required for athletic activity, potentially leading to non-compliance. Return-to-sport criteria generally include restoration of symmetric range of motion with normal strength, as well as the ability to perform sport-specific activities without pain or apprehension. Despite this, high rates of recurrent instability following first-time anterior dislocation is reported, as well as inferior outcomes in patients initially managed nonoperatively who later undergo surgery. 

Management is particularly challenging when injuries occur early or mid-season, as nonoperative treatment enables faster return to play. However, redislocation rates in young athletes undergoing nonoperative management have been reported to be as high as 80–92% [30, 31]. In a prospective randomized study by Bottoni et al., comparing nonoperative treatment with 4 weeks of immobilization versus arthroscopic stabilization for first-time traumatic dislocations in young athletes, 75% of the nonoperative group experienced recurrent instability compared to 11.1% in the operative group [32]. Dickens et al. evaluated return to sport and recurrence rates for anterior shoulder instability in 45 in-season competitive NCAA contact athletes [33]. The authors reported that 73% of athletes returned to play at a median of five days from injury, with 67% completing the season, while only 27% completed the season without a recurrent instability [33]. For in-season athletes, decision-making involves an in-depth discussion of the risks and benefits of nonoperative versus operative management, involving the athlete, coach, trainers, parents and physician, among other key stakeholders.

### Operative Management

**Fig. 2:**
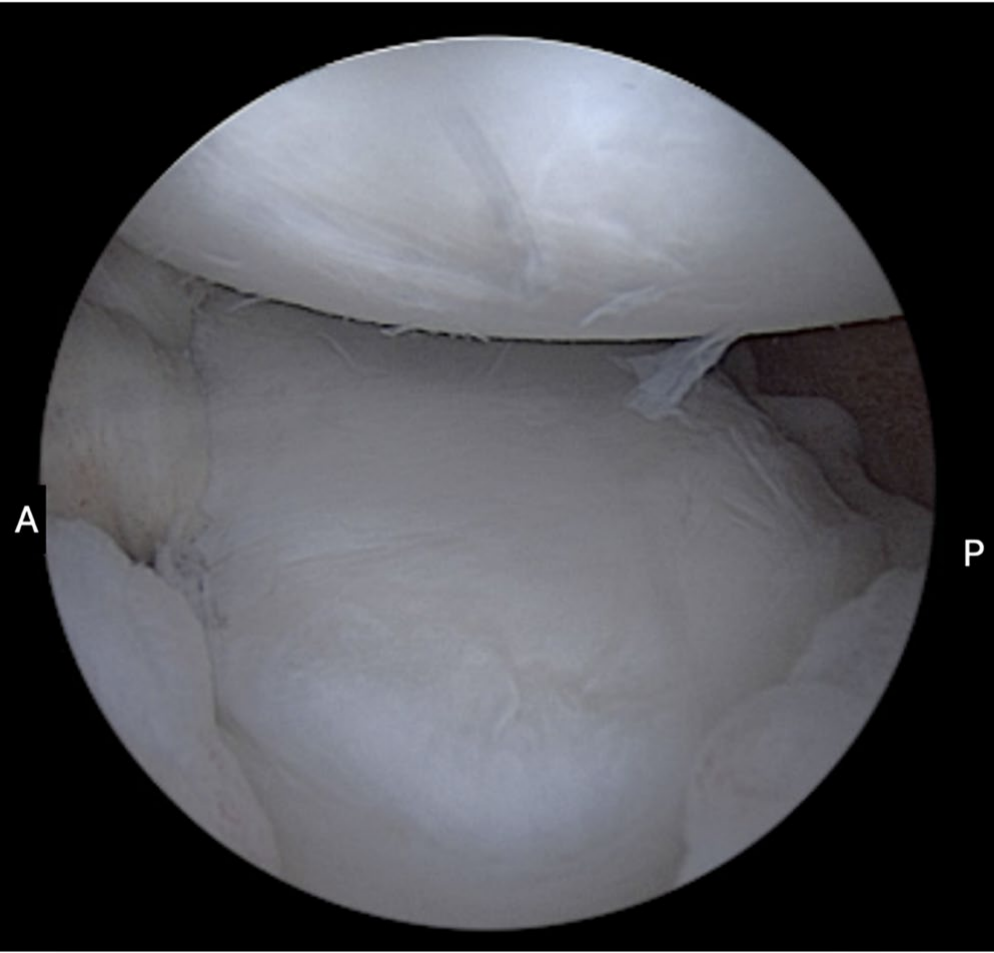
Arthroscopic image of right shoulder anterior labral repair; viewing from the anterosuperior portal in the lateral decubitus position

**Fig. 3:**
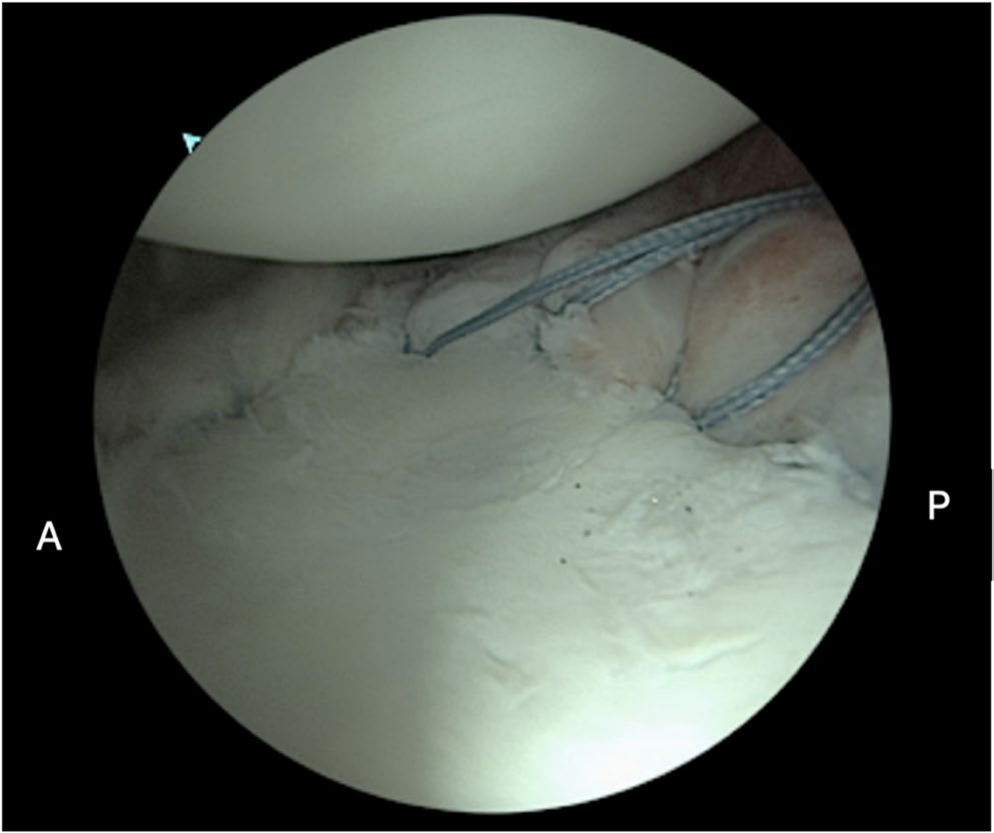
Arthroscopic image of right shoulder posterior labral repair; viewing from the anterosuperior portal in the lateral decubitus position

**Fig. 4:**
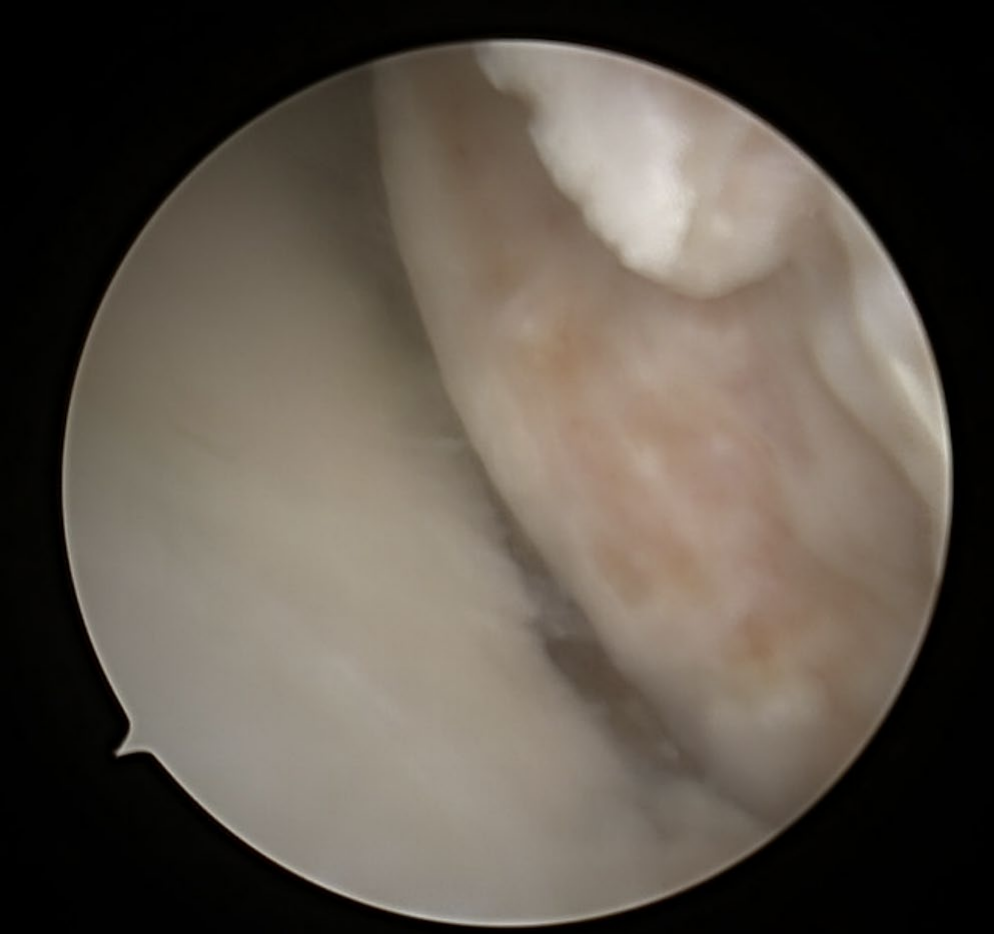
Arthroscopic view from the posterior portal of a right shoulder in beach chair position. Demonstrating dynamic engagement of an off-track large Hill-Sachs lesion with attritional anterior glenoid bone loss

**Fig. 5:**
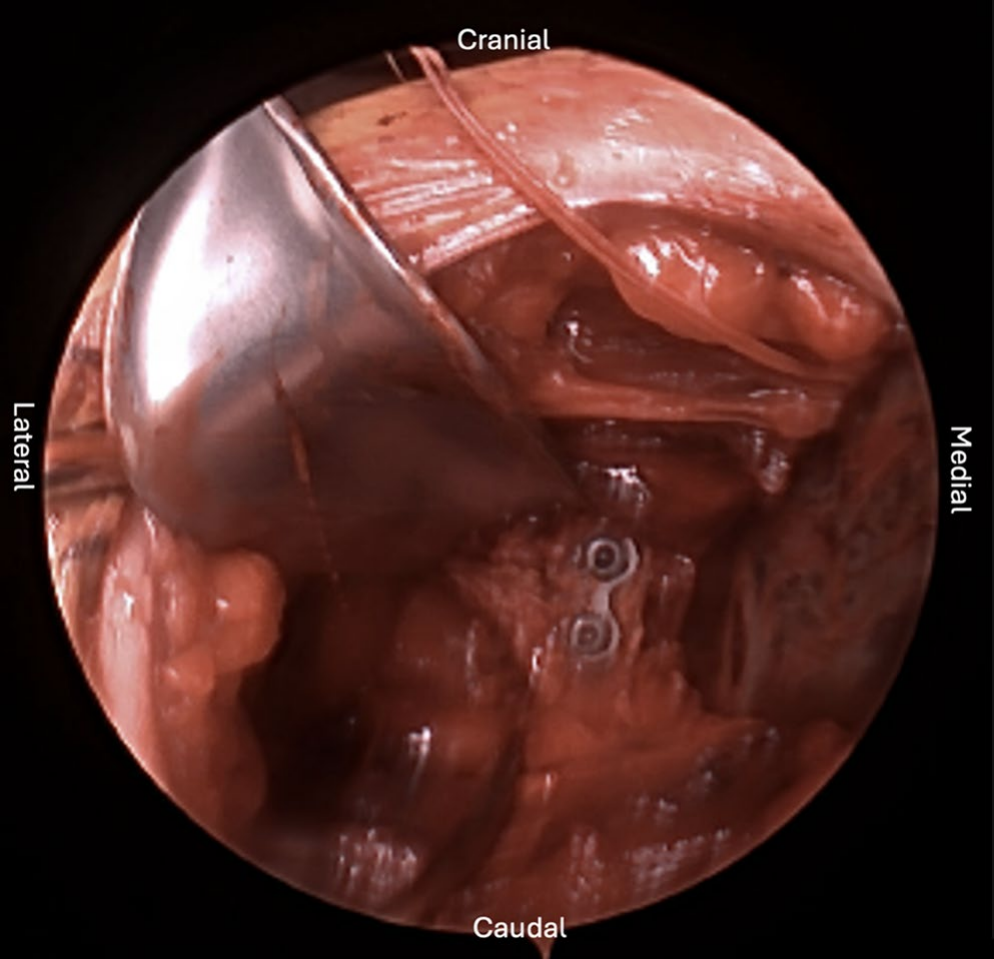
Anterosuperior view of the right shoulder via a deltopectoral approach demonstrating a Latarjet procedure. The transferred coracoid bone block is secured to the anterior inferior glenoid, with the conjoint tendon positioned inferiorly, contributing to dynamic stabilization of the glenohumeral joint

**Fig. 6:**
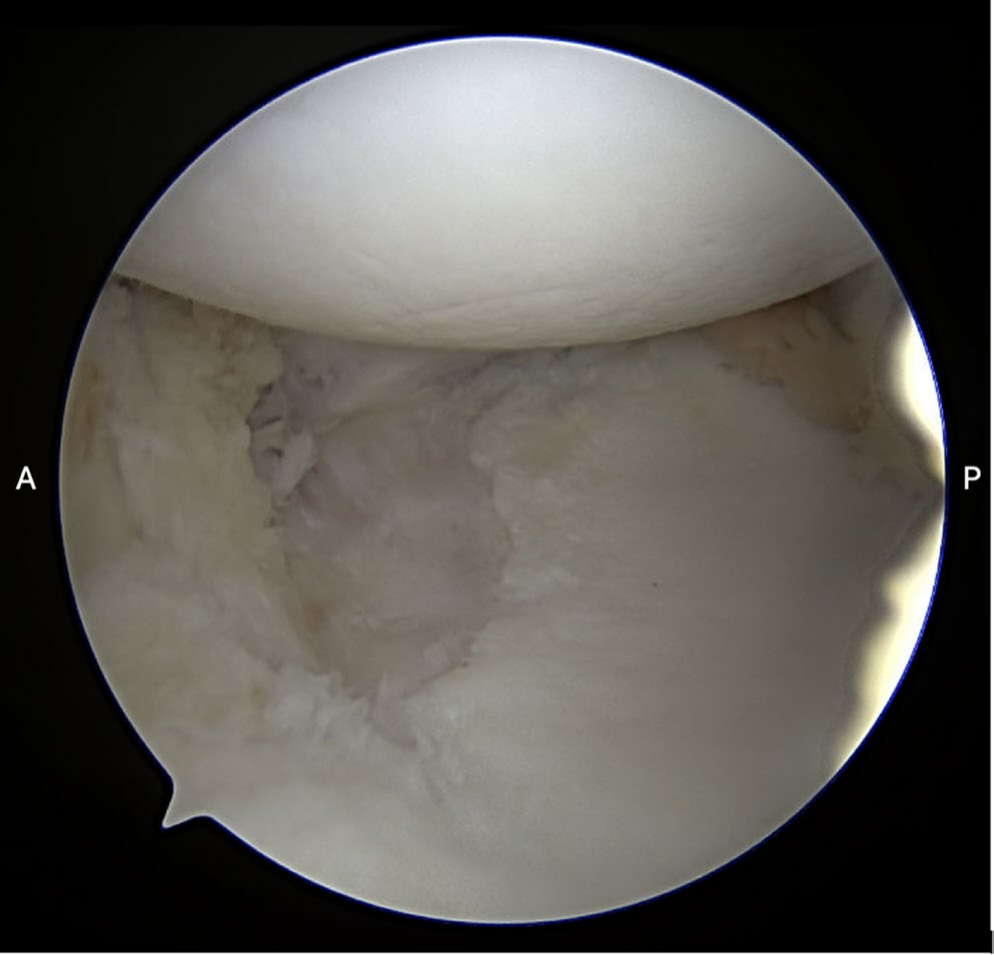
Arthroscopic image of a right shoulder demonstrating graft incorporation of Latarjet with fibrocartilage fill from the anterosuperior portal in the lateral decubitus position

**Fig. 7:**
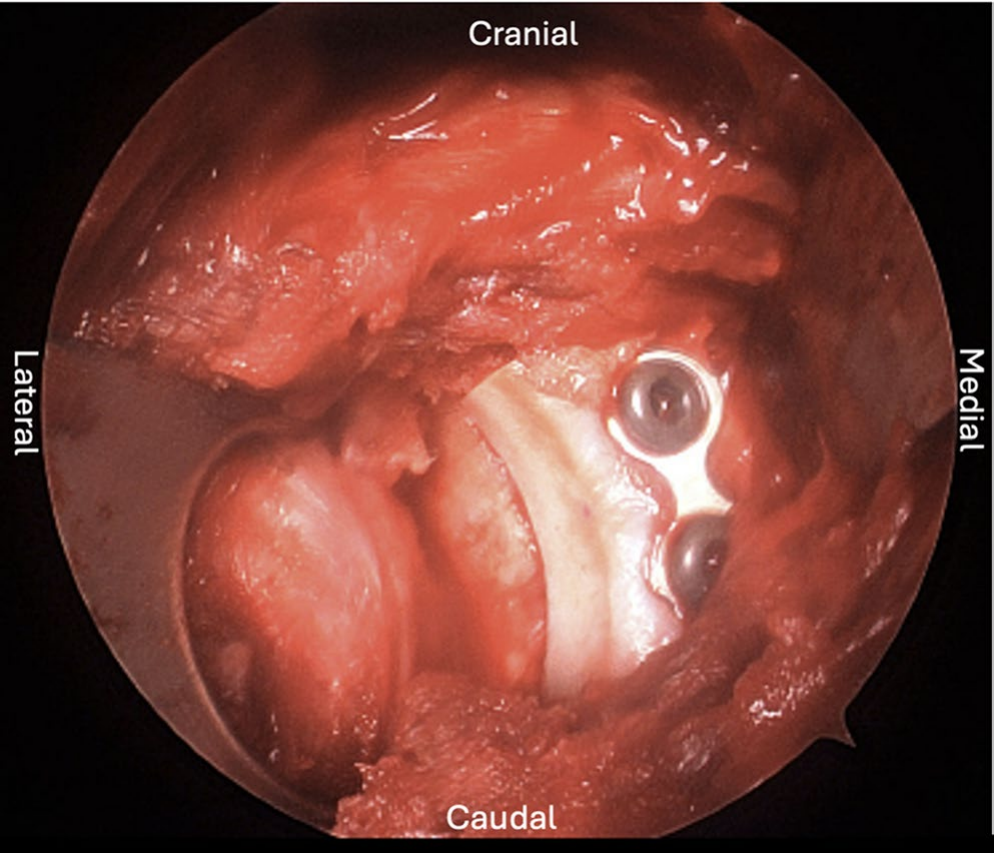
Anterosuperior view of the right shoulder via a deltopectoral approach demonstrating a Distal Tibial Allograft Bone Block Augmentation procedure for anterior shoulder instability with glenoid bone loss

**Fig. 8:**
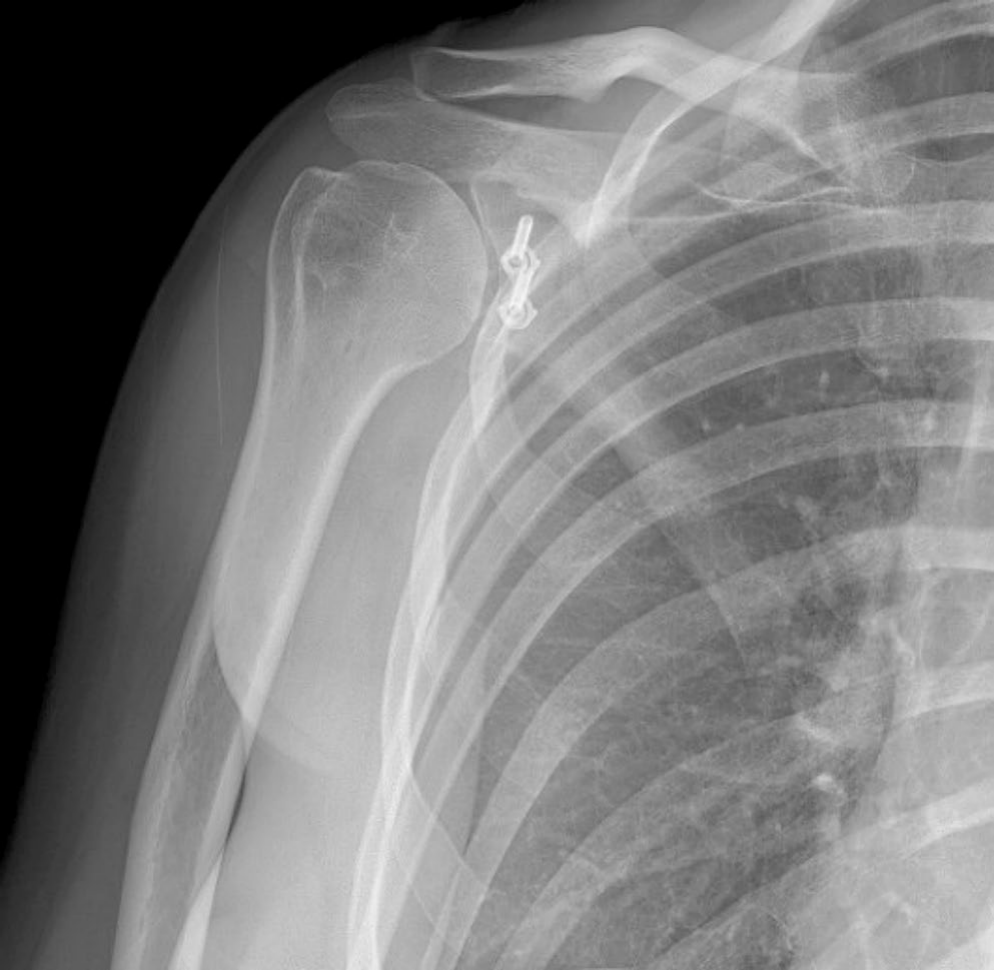
Grashey view of the right shoulder post-operatively after Distal Tibial Allograft Bone Block Augmentation

When considering operative management, it is essential to evaluate for any concurrent pathology and patient-specific factors. The primary goals of stabilization is restoration of stability, range of motion, and strength. Operative management includes arthroscopic labral repair, remplissage, and arthroscopic or open bony augmentation procedures as demonstrated in Figs. 2, 3, 4, 5, 6, 7 and 8. Restoration of the anteroinferior capsulolabral complex and retensioning of the anterior band (and sometimes posterior band) of the IGHL complex are critical. Biomechanical studies have demonstrated restoration of anterior IGHL integrity following arthroscopic Bankart repair [34, 35]. Remplissage, is an adjunct procedure recommended during labral repair or bony restoration to reduce recurrence of instability [36]. The procedure involves the incorporation of the posterior capsule and infraspinatus tendon in a humeral sided Hill-Sachs lesion [36]. While initially indicated as an adjunctive, soft tissue treatment for “off-track” or engaging Hill-Sachs lesions, indications for remplissage have expanded to include “on-track” lesions in selected, higher-risk patients, particularly those with less than 20% glenoid bone, to reduce recurrence rates [37, 38].

**Fig. 9:**
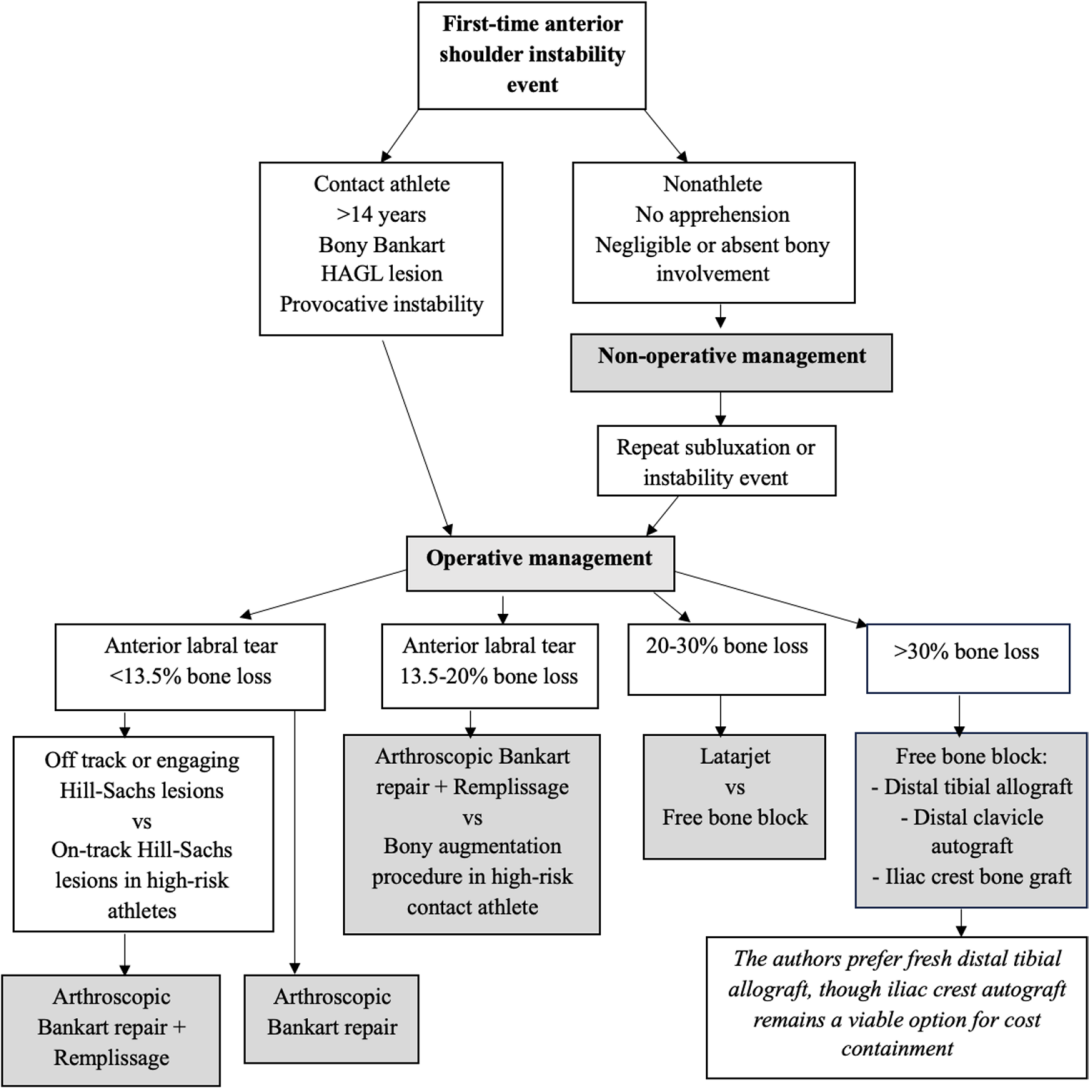
Algorithm for Management of Anterior Shoulder Instability

**Fig. 10:**
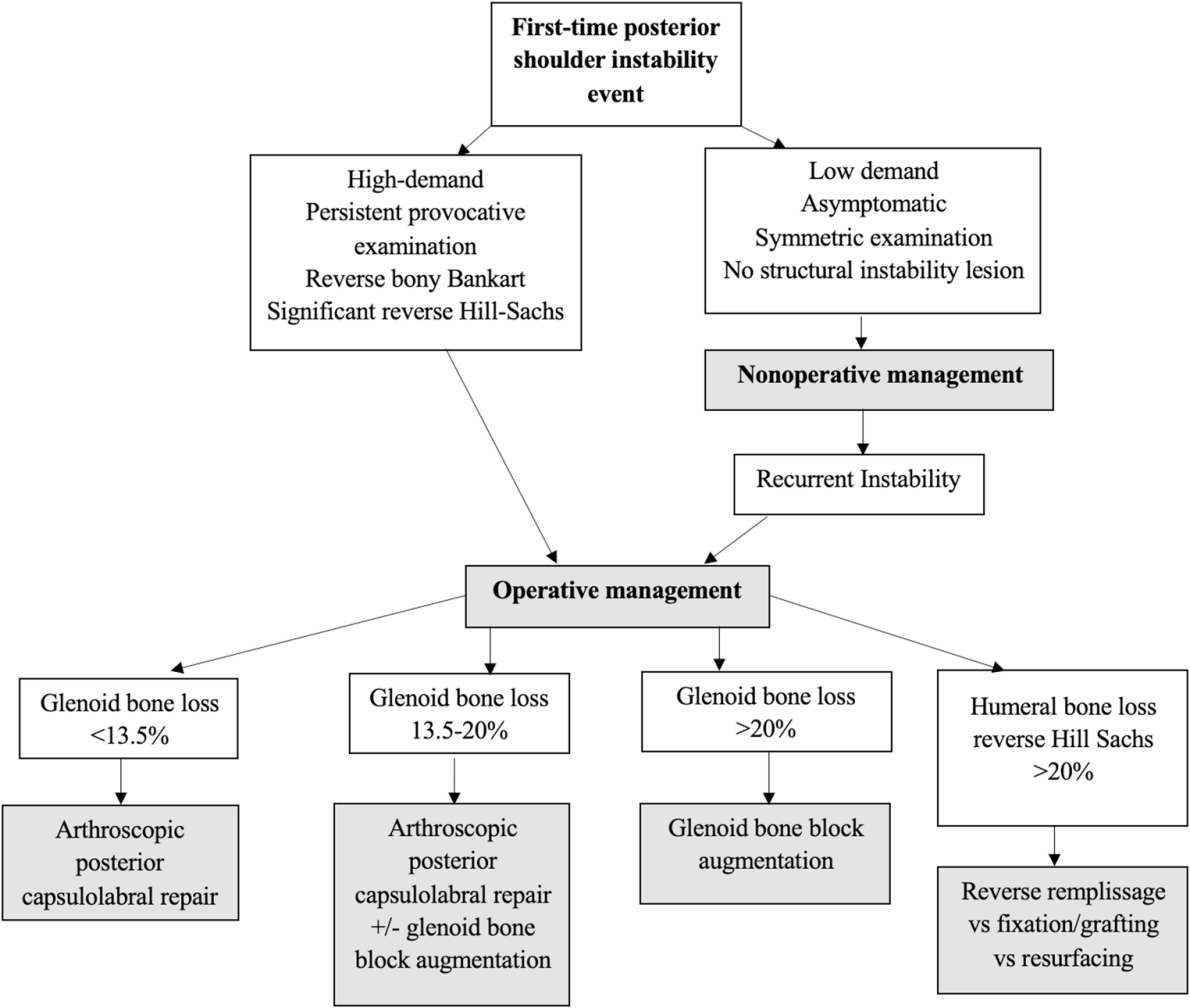
Algorithm for Management of Posterior Shoulder Instability

Labral pathology associated with recurrent glenohumeral instability frequently leads to increasing degrees of attritional bone loss. First-time instability events are associated with an average of 6.8% glenohumeral bone loss, which may increase to 22.8% following recurrent instability events in athletes [39]. Accurate quantification of bone loss and assessment of the glenoid track are essential in determining the need for bony augmentation procedures in addition to labral repair. Critical glenoid bone loss has traditionally been defined as 20–25%, necessitating a bony procedure. Bony procedures include the Latarjet procedure, utilizing a coracoid transfer or free osteochondral bone blocks with distal tibial allograft, distal clavicle, iliac crest or distal radius. Failure rates of isolated arthroscopic Bankart repair in the setting of critical bone loss have been reported to be as high as 75% [39, 40]. However, acceptable glenoid bone loss thresholds have been re-defined, reported as 13.5%-20%, with inferior patient outcomes and high rates of recurrent instability reported in high-risk patients with isolated arthroscopic Bankart repair in the setting of subcritical bone loss [41, 42]. As such, debate regarding lowering the threshold for critical bone loss to between 13.5%, especially in at-risk populations, has been proposed. Based on emerging literature, the authors consider 13.5–15% to represent contemporary critical bone loss among high-risk patients. An algorithm for the management of anterior and posterior shoulder instability is summarized in Fig. 9 and Fig. 10, respectively.

## Rehabilitation

### Nonoperative Management

Rehabilitation following nonoperative management for anterior and posterior shoulder instability typically involves an expedited protocol spanning 2–6 weeks, allowing for expedited return to play. Recovery is generally divided into three phases. The initial phase emphasizes rest, cryotherapy, and anti-inflammatory medications, with the primary goal of achieving painless motion. Early passive motion is initially restricted to the scapular plane at less than 90° of abduction to allow for soft tissue rest [42]. The second phase focuses on stabilization through rotator cuff and periscapular strengthening to restore symmetric strength. Closed-chain exercises are emphasized to encourage coactivation of the rotator cuff, deltoid, and scapular stabilizers. Subscapularis, infraspinatus, and teres minor strengthening is more critical in posterior instability to counteract the deltoid and aid in humeral head compression [42]. The final phase is directed toward returning to sport. Return to play following nonoperative management after anterior shoulder instability has been reported to be as high as 90% in-season athletes; however, approximately 37% are reported to experience recurrent instability during the same season [33].

The standard of care for initial management of MDI is nonoperative, with a 3–6-month rehabilitation program focused on scapulothoracic dyskinesis. This involves strengthening the periscapular and rotator cuff muscles to improve scapular kinematics. Watson and colleagues developed a rehabilitation program for MDI over the course of 3–6 months with initial focus on scapular mechanics, followed by rotator cuff and deltoid strengthening. This six-step protocol consisted of: 1) scapula control and coronal plan control at 0° and 30° abduction 2) posterior musculature development 3) flexion control from 0° to 45° of elevation 4) sagittal plane and coronal plane control from 45° to 90° 5) isolated deltoid drills 6) sports-specific and functional specific [43, 44].

### Operative Management

Rehabilitation following operative management begins with a period of immobilization with a focus on restoring passive and active assisted range of motion for 4–6 weeks, depending on the pathology. Active range of motion and strength training begin at 6 weeks. Plyometrics, push-up activities, and sport-specific training are not initiated until 3 months postoperative for labral repair and until osseous union is confirmed radiographically following bony procedures. At 4.5 months postoperatively, patients can begin to train for return to sport and perform functional assessments. Full return to sport or other at-risk activity should be guided by a regimented return to sport assessment with a skilled physical therapist to reduce the risk of recurrent instability [45]. The senior author’s postoperative rehabilitation protocols for arthroscopic anterior, posterior, and multidirectional shoulder stabilization are summarized in Tables 2 and 3, and 4, respectively. 

Differences in rehabilitation following anterior and posterior stabilization primarily involves avoidance of the at-risk positions in the early phases of rehabilitation—abduction and external rotation in anterior instability versus flexion, adduction, and internal rotation in posterior instability. Following posterior stabilization, an increased focus on subscapularis and infraspinatus strengthening is recommended to promote humeral head compression [44].

Operative management is indicated for MDI after failure of at least 3 consecutive months of rehabilitation. Surgical management for MDI includes arthroscopic capsular plication and open inferior capsular shift, both demonstrating successful outcomes [11]. Thermal capsulorrhaphy can be considered, however this procedure is associated with high failure rates [46]. Postoperative rehabilitation involves 6 weeks of immobilization in a Gunslinger brace held in a neutral position, without any capsular stretching. Caution with overstretching of the capsule is exercised until 3 months postoperatively. After 3 months, patients can begin plyometrics and advance to full range of motion.

**Table 2:** Postoperative rehabilitation protocol for arthroscopic anterior shoulder stabilization

Week 0–1	Sling immobilization, can come out of sling for elbow, wrist, finger ROM
Week 1–4	• Restrict motion to 90° FF, 20° ER at side, IR to stomach, 45° ABD, PROM > AAROM > AROM as tolerated • Hold cross-body adduction until 4 weeks postoperative • Isometric in sling • Sling for 4 weeks
Week 4–8	• Discontinue sling at week 4 • Increase AROM 140° FF, 40° ER at side, 60° ABD IR behind back to waist • Strengthening (isometrics/light bands) within AROM limitations, horizontal abduction exercises • Also start strengthening scapular stabilizers
Week 8–12	• If ROM lacking, increase to full ROM with gentle passive stretching at end ranges • Advance strengthening as tolerated: isometrics > bands > light weights (1–5 lbs.); 8–12 reps/2–3 Strengthen rotator cuff: deltoid, and scapular stabilizers
Months 3–12	• Only do strengthening 3 time a week to avoid rotator cuff tendonitis • Begin upper extremity ergometer • Begin eccentrically resisted motions, plyometrics (weight-ball toss), proprioception (body blade), and closed chain exercises at 12-week mark • Begin sports related rehab at 3 months, including advanced conditioning • Return to throwing progression at 4.5 months • Throw from pitcher’s mound at 6 months

*FF *Forward flexion, *ER *External rotation, *IR *Internal rotation, *ABD* Abduction, *PROM *Passive range of motion, *AAROM *Active assisted range of motion, *AROM* Active range of motion, *PT *Physical therapy, *ROM *Range of motion

**Table 3:** Postoperative rehabilitation protocol for arthroscopic posterior shoulder stabilization

Week 0–3	• Sling in neutral rotation for 3 weeks (padded abduction sling) • Codman exercises, elbow and wrist ROM • Wrist and grip strengthening
Weeks 3–6	• Restrict to FF 90° /IR to stomach PROM - AAROM – AROM ER with arm at side as tolerated • Begin isometrics with arm at side - FF/ER/IR/ABD/ADD • Start scapular motion exercises (trapezius /rhomboids/levator scapulae) • No cross-arm adduction, follow ROM restrictions
Weeks 6–12	• Increase ROM within 20° of opposite side; no manipulations per therapist; encourage patients to work on ROM daily Once 140° active FF, advance strengthening as tolerated: isometrics - bands - light weights (1–5 lbs.); 8–12 reps/2–3 sets per rotator cuff, deltoid, and scapular stabilizers with low abduction angles • Only do strengthening 3x/week to avoid rotator cuff tendonitis • Closed chain exercises
Months 3–12	• Advance to full ROM as tolerated • Begin eccentrically resisted motions, plyometrics (ex. Weighted ball toss), proprioception (ex.body blade) • Begin sports related rehab at 3 months, including advanced conditioning • Return to throwing at 4.5 months • Push-ups at 4.5–6 months • Throw from pitcher’s mound at 6 months

*FF *Forward flexion, *ER *External rotation, *IR *Internal rotation, *ABD *Abduction, *ADD *Adduction, *PROM *Passive range of motion, *AAROM *Active assisted range of motion, *AROM *Active range of motion, *PT *Physical therapy, *ROM *Range of motion

**Table 4:** Postoperative rehabilitation protocol for arthroscopic multidirectional shoulder stabilization with arthroscopic capsular plication

Week 0–6	• Slingshot / Gunslinger Brace for 6 weeks • Isometrics in brace, gentle supported Codman exercises • PROM only for 6 weeks • Grip strengthening, elbow ROM, wrist ROM
Week 6–12	• Sling at night, can discontinue using the sling during the day • AROM only as tolerated to increase ROM; no PT stretching or manipulation • Restrict to 140° FF/ 40° ER at side/ IR to stomach/ 45° Abduction • Scapular stabilization exercises avoiding anterior capsule stress • Begin light isometrics for rotator cuff and deltoid, with arm at the side
Months 3–12	• Advance strengthening as tolerated: isometrics - bands - light weights (1–5 lbs.); 8–12 reps/2–3 set per exercise for rotator cuff; deltoid, and scapular stabilizers • Only do strengthening 3x/week to avoid rotator cuff tendonitis • If ROM lacking, increase to full ROM with gentle passive stretching at end ranges • Begin eccentric motions, plyometrics (ex. Weighted ball toss), and closed chain exercises at 16 weeks • Begin sports related rehab at 4.5 months, including advanced conditioning • Return to throwing at 6 months • Throw from pitcher’s mound at 9 months

*FF *Forward flexion, *ER *External rotation, *IR *Internal rotation, *ABD *Abduction, *ADD *Adduction, *PROM *Passive range of motion, *AAROM *Active assisted range of motion, *AROM *Active range of motion, *PT *Physical therapy, *ROM *Range of motion

### Return to Sport

Maximal medical improvement after arthroscopic anterior and posterior shoulder stabilization is typically 12 months postoperatively. Return to sport criteria are met when range of motion, strength, and endurance are near equivalent to the uninjured shoulder. Athletes must also be able to perform sport-specific drills without pain or apprehension [42].

Psychological readiness is also a critical component in ensuring successful return to play. The Shoulder Instability Return to Sport after Injury (SIRSI) score is a validated tool used to assess psychological readiness following both nonoperative and operative management of shoulder instability. In a prospective cohort study by Rossi et al., patients undergoing arthroscopic Bankart repair or Latarjet augmentation demonstrated a 2.9-fold increased odds of returning to play for every 10-point increase in SIRSI score [47].

## Outcomes

Despite advances in both nonoperative and operative management, recurrent instability remain an area of concern. Increasing evidence suggest improved outcomes following surgical intervention. In a randomized controlled trial by Jakobsen et al., comparing conservative versus open treatment following first-time anterior shoulder dislocation, the recurrence rate at 2-year follow-up was 56% in the nonoperative group versus 3% in the open repair group. At 10-year follow-up, 72% of patients who underwent open repair reported good or excellent outcomes based on the Oxford self-assessment score [48].

Surgical treatment has consistently demonstrated lower rates of recurrent instability at 2 years, with recurrence in 7% compared to 46% in nonoperative cohorts [49]. When comparing surgical techniques, recurrence rates at 2 years were similar between open and arthroscopic approaches (6.4% vs. 8.2%) [49]. In a systematic review by Hurley et al., recurrence rates following first-time dislocation were 9.7% after arthroscopic Bankart repair versus 67.4% following nonoperative management. Return-to-play rates were also greater in the operative group (92.8% vs. 80.8%) [50]. In a systematic review of long-term outcomes after arthroscopic Bankart repair at 10 years follow-up, 77.6% of athletes reported successful return to sport, with an overall recurrent instability of 31.2%, and revision rate of 17% [51].

Arthroscopic stabilization of posterior shoulder instability has also demonstrated favorable return to sport and recurrence rates. At an average of 54 months following stabilization, Pavlik et al. reported a 7.4% recurrence, 98% return to sport activity, with of patients 51% returning to preinjury level [52]. In a systematic review and meta-analysis by Gouveia et al., return to sport and preinjury rate after surgical stabilization for posterior shoulder instability was 88% and 68%, respectively [53]. In contrast, less favorable rates of postoperative instability have been reported after surgical treatment of MDI, up to 29% in patients at 9-year mean follow-up [54]. 

Risk assessment tools have been developed to predict recurrent instability following arthroscopic stabilization. Identified risk factors include near-track lesions, hyperlaxity, younger age, ≥ 2 preoperative instability events, participation in contact sports, increasing glenoid bone loss, and larger Hill-Sachs lesions [37]. A multicenter prospective study of National Collegiate Athletic Association athletes demonstrated that athletes undergoing stabilization after an index instability event were 5.8 times more likely to complete a season without recurrent instability [42]. Additionally, a retrospective analysis of National Football League athletes reported that athletes undergoing stabilization following an initial dislocation had longer career longevity (4.1 seasons vs. 2.8 seasons) when compared to those managed nonoperatively [55].

## Conclusion

Glenohumeral pain and instability due to labral pathology remain a challenging clinical problem. While earlier literature emphasized anterior labral injuries, posterior and combined pathologies are increasingly recognized. Clinical presentation varies by tear type, with posterior tears more commonly presenting with pain and anterior tears more often associated with instability.

Treatment algorithms for labral tears continue to evolve. The decision between nonoperative and operative management should be individualized, along with specific rehabilitation protocols utilized. While nonoperative management may be appropriate for select patients, those with risk factors for recurrence may benefit from early stabilization. 

A patient-specific approach that accounts for patient characteristics, pathology, bone loss, chondral injury, and soft tissue integrity is essential to optimize outcomes.

## Data Availability

No datasets were generated or analysed during the current study.

## Key References

- Waterman BR, Dean RS, Gregory B, Romeo AA. Surgical treatment of superior labral/biceps pathology in the overhead thrower. J Am Acad Orthop Surg. 2023;31.
  - The manuscript provides detailed information on clinical presentation and evaluation of labral pathologies.
- Albertson BS, Trasolini NA, Rue JPH, Waterman BR. In-season management of shoulder instability: How to evaluate, treat, and safely return to Sport. Curr Rev Musculoskelet Med. 2023;16:295–305.
  - The manuscript reports injury rates and treatment outcomes with detailed clinical evaluations and return to sport rates and protocols.
- Charles S, Marcaccio S, Lin RT, Boden S, Nazzal EM, Hughes JD, et al. The Pittsburgh instability tool score predicts outcomes after arthroscopic anterior shoulder stabilization in patients with subcritical bone loss. Arthroscopy. 2025;41(8):e3868–74. doi:10.1016/j.arthro.2025.04.023.
  - The manuscript describes a risk assessment tool created to relate increased bone loss and on-track lesions to outcomes between different surgical techniques.
